# Pharmacokinetics, pharmacodynamics and bioavailability of dexmedetomidine nasal spray in healthy Chinese adults: A phase I clinical trial

**DOI:** 10.3389/fphar.2024.1488462

**Published:** 2024-11-29

**Authors:** Yan Li, Lu Qi, Zhenyu Wang, Wan Wang, Langxi Zhang, Leting Yang, Chen Liu, Wenjing Zhong, Xinghe Wang

**Affiliations:** ^1^ Phase I Clinical Trial Center, Beijing Shijitan Hospital, Capital Medical University, Beijing, China; ^2^ Sichuan Purity Pharmaceutical Co., Ltd., Chengdu, Sichuan, China; ^3^ Chengdu Brilliant Pharmaceutical Co., Ltd., Chengdu, Sichuan, China; ^4^ Chengdu Finelyse Pharmaceutical Technology Co., Ltd., Chengdu, Sichuan, China

**Keywords:** pharmacokinetics, pharmacodynamics, bioavailability, dexmedetomidine, nasal spray

## Abstract

**Background:**

Intranasal administration is a convenient route for drug delivery that can be applied for procedural sedation. However, there is currently limited exploration into fixed dosing regimens. This study was to investigate the pharmacokinetics (PK), pharmacodynamics (PD), bioavailability (BA) and safety of dexmedetomidine after fixed doses of intranasal and intravenous administration in healthy male and female subjects.

**Methods:**

Group A subjects received intranasal or intravenous administration in two periods (12 subjects received intranasal dexmedetomidine (Dex) or the intravenous formulation, and four received the corresponding placebo). Groups B to F underwent single-period dose ascending, receiving only the intranasal Dex formulation or the corresponding placebo (the number of subjects receiving the drug/placebo in groups B to F were 12/2, 12/2, 12/2, 10/2, 10/2, respectively), with doses of 75 μg, 125 μg, 150 μg, 175 μg, and 200 μg, respectively. After administration of each group, blood samples were collected to investigate the plasma concentration of dexmedetomidine, adrenaline and noradrenaline using a HPLC-MS/MS method. Ramsay score, blood pressure and heart rate were collected for safety evaluation. Pharmacokinetic parameters (C_max_, T_max_, AUC_0-24h_, ${\mathrm{AUC}}_{0–\infty }$ , and t_1/2_) of dexmedetomidine were calculated.

**Results:**

A total of 82 subjects were randomized. One subject withdrew for personal reasons before administration and the other subjects completed the entire study process. At a dose of 25 μg, the absolute bioavailability was 59%. Across the dose range of 25 to 200 μg, the median T_max_ was similar (0.5–1 h), and the mean elimination half-life was comparable (3.09–4.28 h), with exposure (C_max_ and AUC_0-t_) increasing with dose. The pharmacokinetics after intranasal spray administration exhibited linear characteristics, although C_max_ was similar in the higher dose groups (175 μg and 200 μg). PD results showed that ideal sedation effects (Ramsay score of 3 or higher in at least 90% of subjects) could be achieved within 30 min following intranasal administration of 75 μg or higher doses. All the subjects were well tolerated without any serious adverse events (SAEs).

**Conclusion:**

Dexmedetomidine nasal spray was well tolerated and achieved satisfactory sedation in the dose range of 25–200 μg in Chinese healthy male and female subjects.

**Clinical Trial Registration:**

http://www.chinadrugtrials.org.cn/, identifier CTR20201650

## Introduction

Dexmedetomidine is a highly selective α2-adrenergic receptor agonist that has been widely used in clinical anesthesia and critical care medicine because of its excellent clinical efficacy, safety, and pharmacodynamics (Bao and Tang, 2020; Penttilä et al., 2004; Roback et al., 2016; Shen et al., 2022). One of the main benefits of dexmedetomidine is its sedative effect, which is achieved through the regulation of the central nervous system activity. The unique mechanism of action of dexmedetomidine, with its selective receptor activity on α2-adrenergic receptors in the locus coeruleus, ensures a preferential sedative effect, with minimal respiratory depression (Goettel et al., 2016; Brede et al., 2004; Gertler et al., 2001). In clinical practice, the use of dexmedetomidine ranges from short-term sedation in the operating room to long-term sedation in critically ill patients in the Intensive Care Unit (ICU). Furthermore, dexmedetomidine has also been used for perioperative analgesia, anti-shivering, prevention of delirium, and reduction of postoperative nausea and vomiting (Yao et al., 2015; Xu et al., 2021). In addition to its use in anesthesia, dexmedetomidine has also shown potential applications in various other fields, including pain management sleep disorders after surgery, fibreoptic nasotracheal intubation and pediatric sedation (Chrysostomou and Schmitt, 2008; Ghai et al., 2017; Zhang et al., 2016; Baier et al., 2016; Tug et al., 2015; Wang et al., 2023; Arora et al., 2024). There is considerable research space for developing indications for pediatric use, such as exploring sedation protocols for preoperative separation anxiety in children and investigating the analgesic and sedative effects before invasive procedures in pediatric patients. Considering ethical aspects, clinical trials for pediatric indications can initially be conducted in healthy adults, followed by extrapolation to children through modeling, and further exploration thereafter.

In terms of pharmacokinetic characteristics, dexmedetomidine is characterized by its rapid absorption and elimination. After intravenous infusion, the half-life (t_1/2_) of the rapid distribution phase of dexmedetomidine is about 6 min. The terminal clearance half-life is about 2 h. The clearance rate is about 39 L/h. Within 24 h of intravenous infusion of dexmedetomidine at a rate of 0.2-0.7 μg/kg/h, dexmedetomidine exhibited linear kinetics. The steady-state distribution volume (V_ss_) of dexmedetomidine is approximately 118 L. The average protein-binding rate of dexmedetomidine is 94% (Yoo et al., 2015; Weerink et al., 2017; Li et al., 2018). Dexmedetomidine undergoes hepatic metabolism primarily via cytochrome P450 enzymes (Wang et al., 2018). The major metabolite of dexmedetomidine is 3-hydroxy-dexmedetomidine, which is less active than the parent compound. The vast majority of administered dexmedetomidine is eliminated in the urine, and very few are excreted in feces (Weerink et al., 2017; Kivistö et al., 1994).

Dexmedetomidine hydrochloride injection was first developed by Orion Pharma (Finland) and Abbott (USA). It was approved by the FDA on 17 December 1999, under the trade name Precedex^®^. Currently, the injection formulations of dexmedetomidine hydrochloride are widely used. However, intranasal administration is a convenient and rapid method of administration when used for preoperative induction anesthesia, endoscopy, and children’s preoperative separation anxiety. Therefore, the development of nasal sprays is of great clinical value. Particularly, to achieve convenient fixed-dose intranasal administration, further investigation is needed into its relative bioavailability and appropriate fixed doses.

Based on the above background, we conducted this study to evaluate the safety, tolerability, pharmacokinetics (PK) and pharmacodynamics (PD) of dexmedetomidine in healthy subjects after an ascending dose of dexmedetomidine nasal spray and investigated the bioavailability (BA) of dexmedetomidine. This study aims to explore the Dex doses that can be tolerated by adults to confirm the fixed doses selectable for Phase II studies, with the goal of achieving convenient fixed-dose administration in the future. Additionally, by examining the PK parameters at different doses, we can provide theoretical foundations for subsequent modeling and extrapolation to pediatric studies.

## Methods

### Ethics

This study was performed at the Beijing Shijitan Hospital, Beijing, China. The study protocol and informed consent to participate were reviewed and approved by the Institutional Review Board of the Beijing Shijitan Hospital (2020 (40)). The study was performed in accordance with consensus ethics principles derived from international ethics guidelines, including the Declaration of Helsinki, the International Council for Harmonization Guidelines for Good Clinical Practice (GCP), and all applicable laws, rules, and regulations.

### Formulations

The dexmedetomidine nasal spray formulation was obtained from Sichuan Purity Pharmaceutical Co., Ltd., Sichuan, China (1 mL: 500 μg, 25 μg/spray, batch number: CP033.2-201904, expiry date: 19 Sep 2021). The injection formulation was from Yangtze River Pharmaceutical Group Co., Ltd, Jiangsu, China (2 mL: 0.2 mg, batch number: 19,121,931, expiry date: November 2021). A placebo with the same appearance as the dexmedetomidine nasal spray and injection was also provided.

### Subjects

Healthy Chinese male and female volunteers aged 18–65 years were certified as healthy based on a comprehensive clinical assessment that included a detailed medical history, comprehensive physical examination, vital signs, electrocardiogram, and laboratory parameters. Female subjects of childbearing age were required to have negative results on a pregnancy test, and only those who agreed to use an appropriate method of contraception during the study period were included. Subjects with nasal diseases, lung diseases, or allergies to sedatives were excluded. Participants were restricted to the use of concomitant medications, tobacco, alcohol, and food supplements throughout the study.

### Study design and treatment

A randomized, single-center, randomized, double-blind, placebo-controlled, dose-ascending study (Study Number: CTR20201650, http://www.chinadrugtrials.org.cn) was conducted in 82 healthy Chinese subjects (half male and half female) at Beijing Shijitan Hospital (Capital Medical University, Beijing, China).

This study consisted of six groups. The study was randomized and double-blinded. Group A subjects received intranasal or intravenous administration in two periods (12 subjects received intranasal dexmedetomidine (Dex) or the intravenous formulation, and four received the corresponding placebo). Groups B to F underwent single-period dose ascending, receiving only the intranasal Dex formulation or the corresponding placebo (the number of subjects receiving the drug/placebo in groups B to F were 12/2, 12/2, 12/2, 10/2, 10/2, respectively), with doses of 75 μg, 125 μg, 150 μg, 175 μg, and 200 μg, respectively. No food was allowed for a minimum of 4 h after administration. On the days of administration, standard lunch and dinner were given at least 4 and 10 h after administration, respectively.

### Blood sample collection and PK and PD analysis

#### PK parameters

In each group, about 4 mL of venous blood was collected at the following 16 time points: 0 h (within 1 h before administration), 5 min, 10 min, 15 min, 20 min, 30 min, 45 min, 1 h, 1.5 h, 2 h, 3 h, 4 h, 6 h, 8 h, 10 h, 12 h and 24 h after administration for PK analysis. The primary PK parameters were the peak concentration (C_max_), the area under the concentration-time curve from time 0 to the last measurable concentration time point (AUC_0–t_), and the AUC from time 0 to infinity ( ${\mathrm{AUC}}_{0–\infty }$ ) of dexmedetomidine. The observed time to C_max_ (T_max_), t_1/2_ (half-life time), elimination rate constant (λ_z_), and absolute bioavailability (Fabs) were secondary parameters.

#### PD Parameters.

The Ramsay score, heart rate, blood pressure, adrenaline and plasma noradrenaline concentrations were measured to assess the pharmacodynamic effects of dexmedetomidine.

For adrenaline and noradrenaline concentration analyses, about 4 mL of venous blood was collected at the following 11 time points: 0 h (within 1 h before administration), 5 min, 10 min, 15 min, 20 min, 30 min, 45 min, 1 h, 1.5 h, 2 h, and 3 h after administration. Ethylene diamine tetra acetate (EDTA) was used as the anticoagulant. The Ramsay score was assessed at the same time points.

Blood pressure and heart rate (pulse) were measured 0 h before administration (within 1 h before administration) and at 5, 10, 15, 20, 30, 45 min, 1 h, 1.5 h, 2 h, 3 h, 4 h, 6 h, 8 h, 10 h,12 h and 24 h after administration.

#### PK and PD analysis

Plasma concentrations of dexmedetomidine, adrenaline and noradrenaline were determined using HPLC-MS/MS. Blood samples were centrifuged at 2°C–8°C and 1,700 g for 10 min. After centrifugation, the plasma samples were divided into two parts: one tube was used for content determination (>0.8 mL), and the remaining plasma was placed in another tube as a backup. All samples were centrifuged within 30 min after collection. Plasma samples were temporarily stored in a freezer at approximately −20 °C and then moved to another freezer and kept at −60 to −90 °C for long-term storage before pharmacokinetic analysis.

After blood samples were collected from all subjects, plasma samples for testing were transferred to Chengdu Finelyse Pharmaceutical Technology Co., Ltd (Chengdu, Sichuan, China) for bioanalysis using a previously fully validated HPLC-MS/MS bioanalytical method for the quantitation of dexmedetomidine. A Shimadzu LC-30AD HPLC system (Shimadzu, Kyoto, Japan) was coupled with an AB Sciex 5500 Triple Quadrupole MS/MS instrument (Concord, Ontario, Canada) equipped with an electrospray ionization interface in the positive ion and multiple reaction monitoring modes. Analyst 1.6.3 software was used for data acquisition and peak integration. Watson^®^ LIMS software (version 7.5) was used for the sample management and regression calculation. The method was fully validated according to the Chinese Pharmacopeia 9012 Quantitative Bioanalytical Method Validation Guidelines (2015) and the US FDA Bioanalytical Method Validation Guideline for Industry (May 2018). The recovery was 76.58%–96.64%. The interday precision (coefficient of variation (CV %)) was <4.91%, and the accuracy ranged within −1.57%–6.47%. The linearity range for dexmedetomidine, adrenaline and noradrenaline was 3.500–2000 pg/mL, 5.000–500.0 pg/mL, 20.00–800.0 pg/mL, respectively.

### Safety assessment

The subjects were carefully monitored for vital signs, SpO_2_, physical examinations, laboratory parameters (hematology, biochemistry, and urinalysis), and standard 12-lead electrocardiogram (ECG). The measurement of blood pressure and heart rate (pulse) were explained in the previous paragraphs. SpO_2_ and ECG were measured before administration, and also 2 h and 4 h after administration. Adverse events were graded according to Common Terminology Criteria for Adverse Events version 5.0 and classified according to System Organ Class or Preferred Term according to the Medical Dictionary for Regulatory Activities (version 22.0).

### Statistical analysis

PK analysis was conducted in randomized subjects with at least one evaluable PK parameter (pharmacokinetic parameter set, PKPS). Prior to the analysis, all values below the lower limit of quantification were recorded as zero when calculating the mean plasma concentrations. The arithmetic mean, standard deviation, coefficient of variation, median, maximum and minimum values of the parameters were presented. The pharmacokinetic parameters, T_max_, C_max_, AUC_0-t_, ${\mathrm{AUC}}_{0–\infty }$ , V_z_, t_1/2z_, MRT, CL and F for each subject were calculated using a non atrioventricular model based on PKPS by non-compartment modeling using Phoenix WinNonlin version 8.4 (Pharsight Corporation, Mountain View, California). Other analyses were performed by using SAS version 9.4 (SAS Institute Inc., Cary, North Carolina). The PD parameter analysis of adrenaline and noradrenaline was also carried out in the same way. The relationship between AUC_0-t_, ${\mathrm{AUC}}_{0–\infty }$ , C_max_ and dose of intranasal dexmedetomidine (Dose proportional relationship analysis, linear or nonlinear) was analyzed by power function model, and the linear criterion was 80.00%–125.00%.

## Results

### Participants

A total of 82 subjects were enrolled in the study (16 enrolled in Group A and 66 enrolled in Group B to F). Of these, one subject dropped out spontaneously before the administration because of personal reasons. The remaining 81 subjects completed the whole procedure. Among the 81 subjects, 41 were male and 40 were female. For the baseline data of subjects, there was no significant difference in age, height, weight and BMI between the dexmedetomidine group and the placebo group. The demographics and subject characteristics at baseline are presented in Supplementary Table S1. The subjects disposition flow diagram is presented in Supplementary Figure S1.

### Bioavailability and pharmacokinetics

**FIGURE 1:**
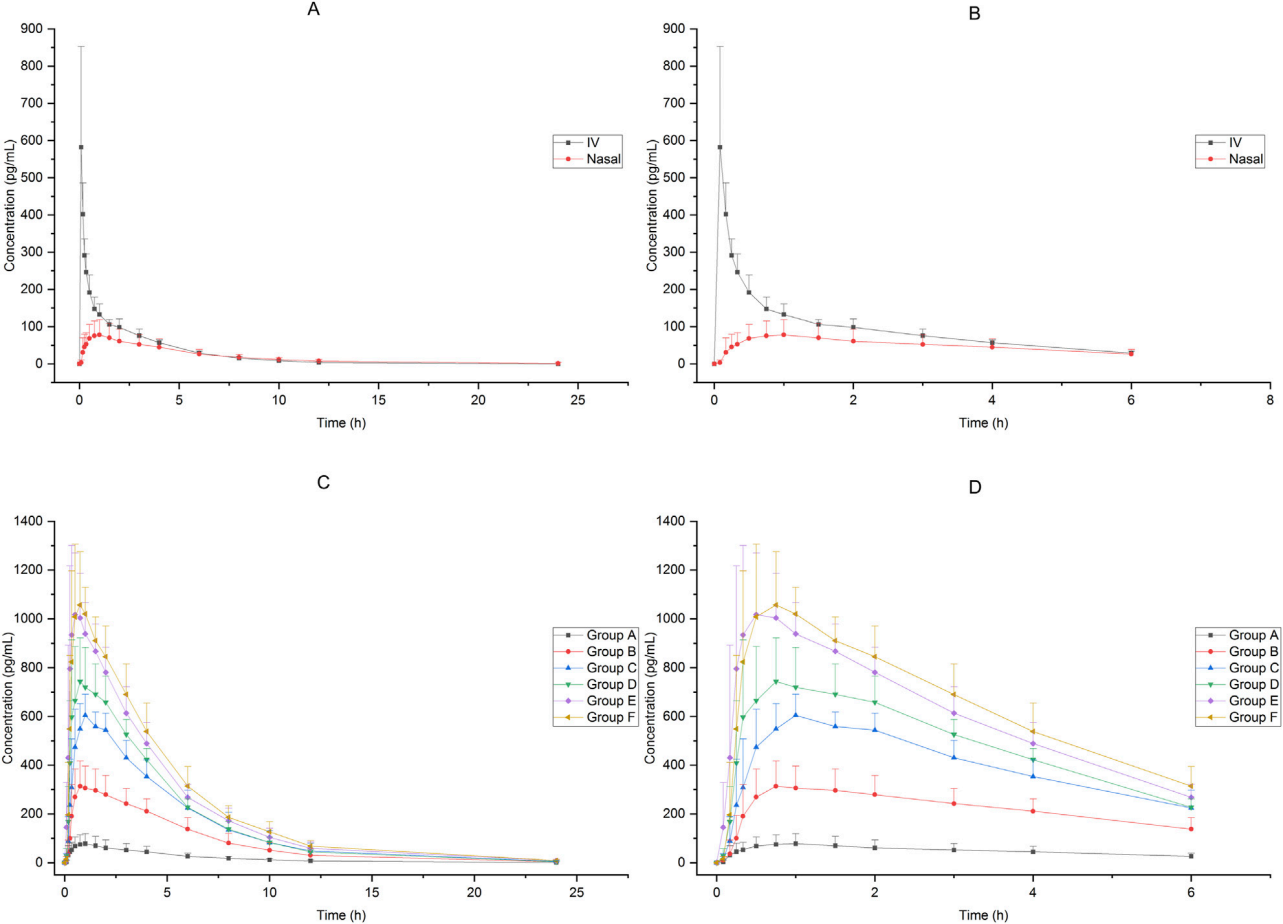
Mean blood concentration-time profiles after receiving 25μg−200 μg dexmedetomidine **(A)**:25 μg IV vs. 25 μg nasal spray(0−24 h), **(B)** 25 μg IV vs. 25 μg nasal spray(0−8 h),**(C)**: 25μg−200 μg nasal spray(0−24 h), **(D)** 25μg−200 μg nasal spray(0−8 h)).

In Group A, the absolute bioavailability of nasal spray administration versus intravenous administration was assessed in 12 subjects who received dexmedetomidine. The pharmacokinetic data for the two formulations are summarized in Table 1. The nasal spray formulation has an estimated bioavailability of 59%. The mean blood concentration-time profiles of dexmedetomidine in Group A are shown in Figures 1A, B.

**TABLE 1:** Pharmacokinetic parameters (mean ± SD) for plasma concentrations of dexmedetomidine after receiving 25 μg dexmedetomidine nasal spray and injection.

Parameters	Group A (25 μg)	
	Nasal spray(n = 12)	IV(n = 11)^a^
T_max_(h)^b^	0.75(0.17,4.00)	0.08(0.08,0.17)
C_max_(pg/mL)	85.14 ± 42.08	611.78 ± 235.83
AUC_0-t_(h^b^pg/mL)	408.53 ± 169.68	658.79 ± 87.88
AUC_0-∞_(h^b^pg/mL)	455.19 ± 167.87	675.88 ± 90.34
AUC_%Extrap_(%)	11.08 ± 7.05	2.58 ± 0.92
λ_z_(1/h)	0.19 ± 0.07	0.32 ± 0.05
t_1/2z_(h)	4.28 ± 2.00	2.24 ± 0.37
Fabs	—	0.59 ± 0.31

^b^ T_max_ was represented by the median (minimum, maximum).

^a^ The blood concentration data of one subject in the intravenous administration period was excluded, because at one time point the blood was taken from the indwelling needle for intravenous infusion of dexmedetomidine and the blood concentration of that time point was much higher than the others.

Sixty-six subjects received nasal spray formulation from Group A to Group F (56 of them received dexmedetomidine and the other 10 received placebo; one subject withdrew before administration for personal reasons). The mean blood concentration-time profiles of dexmedetomidine are shown in Figures 1C, D. The PK parameters of the subjects are summarized in Table 2.

**TABLE 2:** Pharmacokinetic parameters (mean ± SD) for plasma concentrations of dexmedetomidine after ascending single nasal-spray administration.

Parameters	Group A (25μg, IV) (n = 11)	Group A (25μg, nasal spray) (n = 12)	Group B(75 μg) (n = 12)	Group C(125 μg) (n = 11)^and^	Group D(150 μg) (n = 12)	Group E(175 μg) (n = 10)	Group F(200 μg) (n = 10)
T_max_(h)^a^	0.08(0.08,0.17)	0.75(0.17,4.00)	1.00(0.50,6.00)	1.00(0.50,2.00)	0.75(0.33,1.50)	0.50(0.25,2.00)	0.75(0.33,2.00)
C_max_(pg/mL)	611.78 ± 235.83	85.14 ± 42.08	342.23 ± 89.22	632.10 ± 87.37	809.43 ± 204.73	1112.47 ± 262.22	1119.06 ± 200.27
AUC_0-t_(h^a^pg/mL)	658.79 ± 87.88	408.53 ± 169.68	1903.98 ± 549.16	3404.69 ± 756.68	3893.94 ± 609.27	4966.48 ± 660.25	5395.86 ± 940.11
AUC_0-∞_(h^a^pg/mL)	675.88 ± 90.34	455.19 ± 167.87	1977.27 ± 591.38	3448.52 ± 751.14	3943.00 ± 590.15	5014.35 ± 678.57	5440.67 ± 953.10
AUC_%Extrap_(%)	2.58 ± 0.92	11.08 ± 7.05	3.52 ± 2.36	1.36 ± 0.94	1.36 ± 1.54	0.93 ± 0.53	0.80 ± 0.28
λ_z_(1/h)	0.32 ± 0.05	0.19 ± 0.07	0.25 ± 0.08	0.22 ± 0.05	0.24 ± 0.06	0.19 ± 0.03	0.20 ± 0.02
t_1/2z_(h)	2.24 ± 0.37	4.28 ± 2.00	3.22 ± 1.32	3.35 ± 0.72	3.09 ± 0.68	3.74 ± 0.59	3.47 ± 0.32

^a^ T_max_ was represented by the median (minimum, maximum).

^and^ One subject withdrew before administration.

### Pharmacodynamics

#### Ramsay score

**FIGURE 2:**
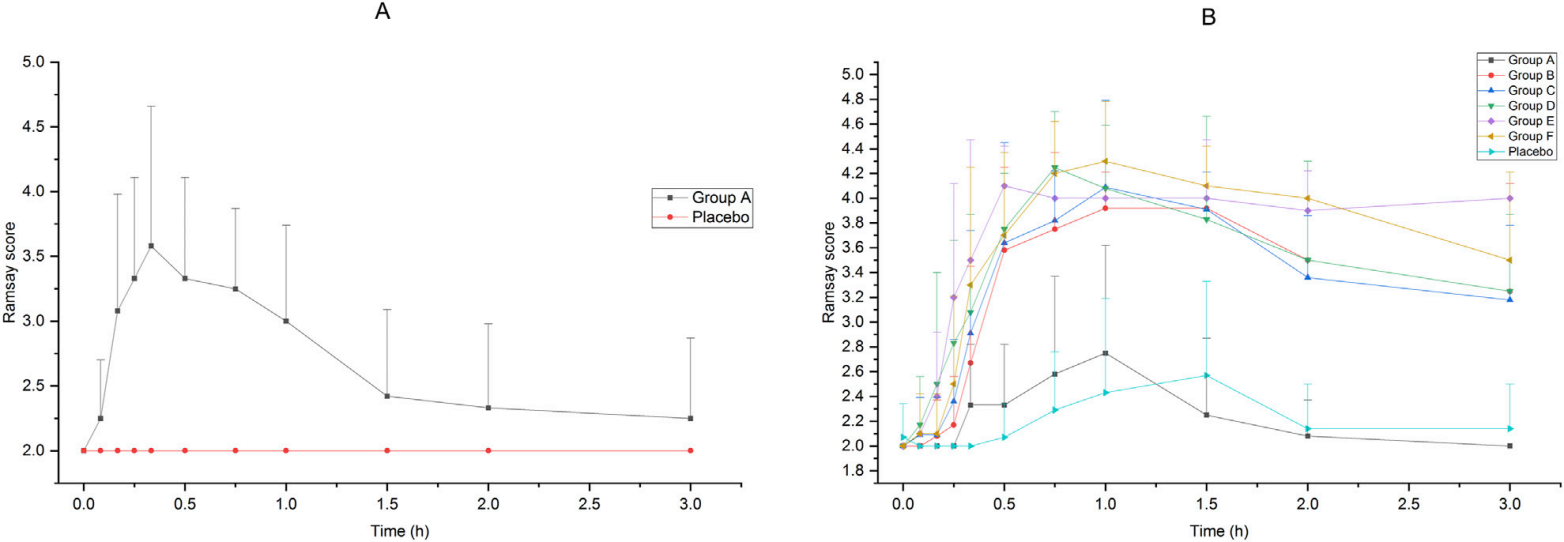
Ramsay Score of subjects accepted 25μg−200 μg dexmedetomidine nasal spray, injection or placebo **(A)**:25 μg IV vs. placebo, **(B)**25μg−200 μg nasal spray vs. placebo).

The Ramsay scores of the subjects who received intranasal or intravenous administration in Groups A to F are shown in Figure 2. Compared with intravenous administration, it took a longer time for intranasal administration of dexmedetomidine to achieve a satisfactory sedative effect, but the effect lasted longer. After receiving 25 μg intravenous administration of dexmedetomidine, the Ramsay score reached 3 in 15 min, which indicated an ideal sedative state, and it could last about 1 h after administration. After receiving 75 μg–200 μg of intranasal administration of dexmedetomidine, the Ramsay scores maintained at 3 or above from 30 min to 3 h. Accepting 25 μg of dexmedetomidine nasal spray or placebo cannot achieve ideal sedation.

#### Adrenaline and noradrenaline

**FIGURE 3:**
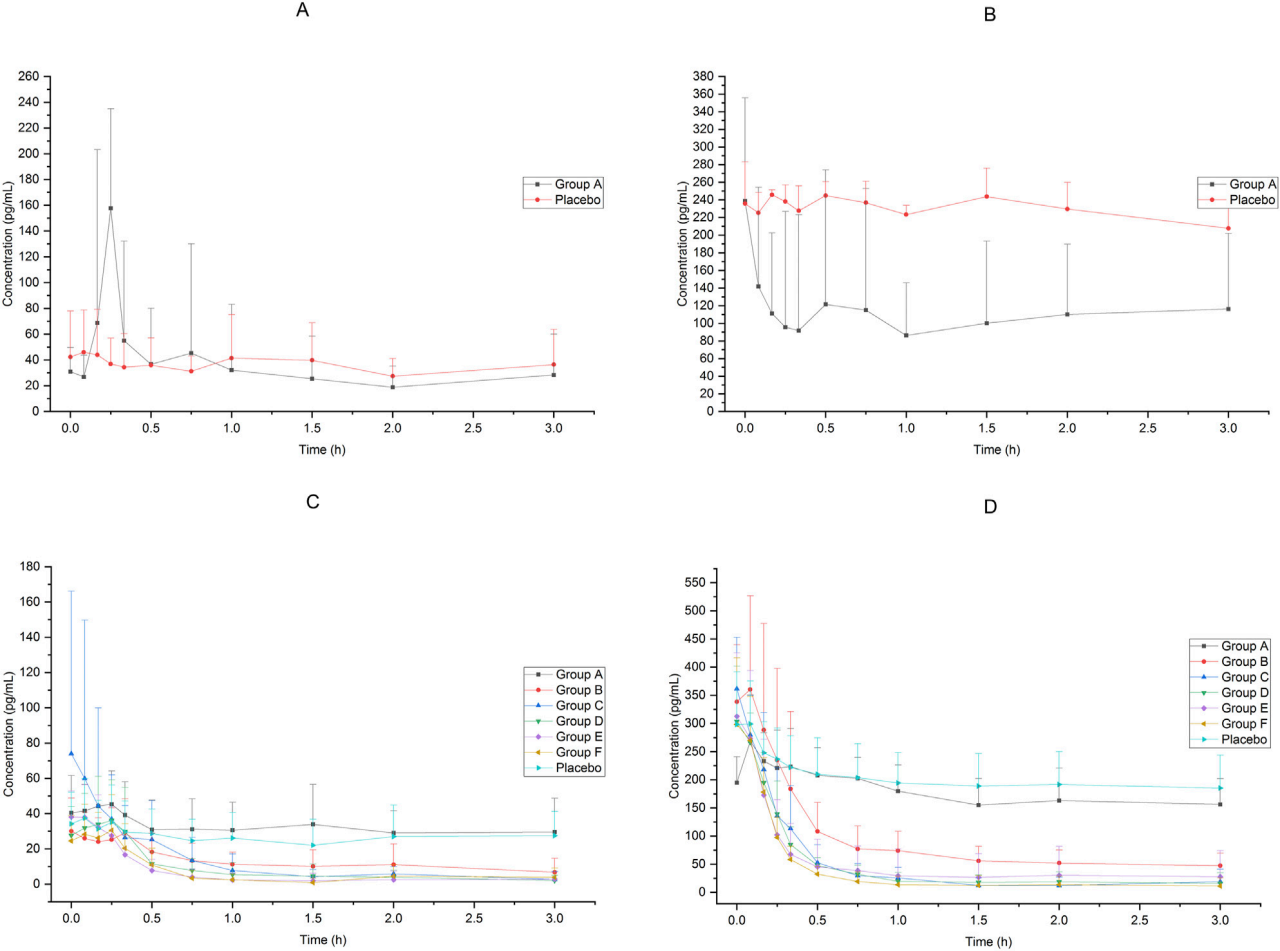
Adrenaline and noradrenaline concentration of subjects accepted 25μg−200 μg dexmedetomidine nasal spray, injection or placebo **(A)**: adrenaline, 25 μg IV vs. placebo, **(B)** noradrenaline, 25 μg IV vs. placebo, **(C)** adrenaline, 25μg−200 μg nasal spray vs. placebo, **(D)** noradrenaline, 25μg−200 μg nasal spray vs. placebo).

Changes in adrenaline and noradrenaline concentrations after dexmedetomidine nasal spray administration were similar to those in Ramsay’s score. The changes of adrenaline and noradrenaline concentration in Group A were not significant within 3 h after administration, and the concentrations of adrenaline and noradrenaline in Groups B to F decreased significantly from 30 min after administration, and could lasted up to 3 h after administration (the last collection time point). The results are shown in Figure 3.

#### Heart rate and blood pressure

**FIGURE 4:**
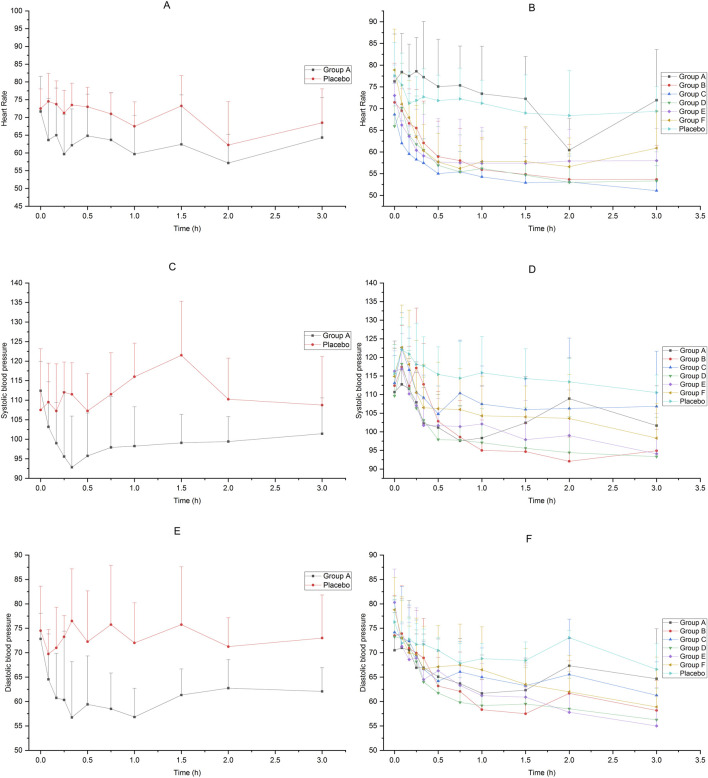
Heart rate and blood pressure of subjects accepted 25μg−200 μg dexmedetomidine nasal spray, injection or placebo **(A)**: heart rate, 25 μg IV vs. placebo, **(B)** heart rate, 25μg−200 μg nasal spray vs. placebo, **(C)** systolic pressure, 25 μg IV vs. placebo, **(D)** systolic pressure, 25μg−200 μg nasal spray vs. placebo, **(E)** diastolic pressure, 25 μg IV vs. placebo, **(F)** diastolic pressure, 25μg−200 μg nasal spray vs. placebo).

The heart rate and blood pressure of the subjects in each group decreased after receiving dexmedetomidine compared to placebo. The average change rate of diastolic blood pressure in Group E decreased by more than 30% from 3 h to 6 h after administration, and the average change rate of diastolic blood pressure in Group F decreased by more than 30% at 6 h after administration. The blood pressure at other time points in each groups did not drop by more than 30%. The results are shown in Figure 4.

### Safety

In Group A after receiving 25 μg of dexmedetomidine injection, 11 subjects (91.67%, 11/12) experienced 35 adverse events, of which 34 were classified as adverse reactions. The severity of three AEs (low blood pressure) was grade 3, whereas that of the remaining AEs was grades 1–2. In Groups A−F, after receiving 25–200 μg of dexmedetomidine nasal spray, 65 subjects (97.01%, 65/67) experienced 237 adverse events, of which 231 were classified as adverse reactions. The severity of four AEs (two cases of low heart rate, one case of syncope, and one case of hypoxia) was grade 3, whereas that of the remaining AEs was grades 1–2. All subjects completely recovered or improved. There were no serious AEs in the whole study. The details are shown in Supplementary Tables S2−S4. The 25–200 μg dose of dexmedetomidine nasal spray and 25 μg dose of dexmedetomidine injection were generally safe and well tolerated.

## Discussion

This study is a dose-ascending study aimed to evaluate the pharmacokinetics and pharmacodynamics of dexmedetomidine nasal spray in healthy Chinese male and female adults. A previous study (Kuang et al., 2022) including dose groups of 20, 40, 100, and 150 μg demonstrated that these doses were tolerable in healthy adults. Compared with Kuang’s study, our study explored more dose groups and a higher maximum dose. Our study verified that there was a good dose-effect relationship in healthy Chinese adults after administration of dexmedetomidine nasal spray, and the C_max_ and AUC were positively proportional to the ascending dose of 25–175 μg. At the dose of 200 μg, the C_max_ and AUC were similar to those at the dose of 175 μg, suggesting that the nasal absorption rate of this product was close to saturation when it was above 175 μg (7 sprays). In another trial studying the PK and PD characters of an 84 μg dose of dexmedetomidine nasal spray in healthy Caucasian adults (Iirola et al., 2011), the C_max_ and AUC were similar to that of 75 μg dose in our study. Furthermore, the BA in that study was 65%, which was also very close to that in our study (59%). A study (Li et al., 2018) using weight-based dosing (1 μg/kg) indicated a BA of about 40%, while the model-calculated BA was approximately 50%. The BA of our study is between these two studies, which provides a new theoretical basis for the BA of Dex.

This study explored the tolerance of dexmedetomidine administration with fixed dose, rather than determining the dosage according to body weight. Each shot of nasal spray contains 25 μg of dexmedetomidine. The results show that the dose range of 25–200 μg is tolerable for the subjects, that is, 1–8 shots of nasal spray are safe. According to the PD and safety data of this study, 75 µg and above doses of nasal spray can achieve an ideal sedative effect 30min after administration (at least 90% of the subjects could reach a Ramsay score of 3), and at the same time, more than 125 µg will lead to the safety risk of diastolic blood pressure decreasing by more than 30% relative to the baseline value (Groups E and F). A dose range of 75–125 µg is recommended for phase II trials. A study (Gao et al., 2024) in children showed that fixed doses of 30 or 50 μg administered intranasally could achieve sedation scores of ≥3 and facilitate parent-child separation. The PD results of this study also support adequate sedation duration and depth, allowing for further exploration of longer-lasting sedation effects in subsequent studies.

Compared with intravenous administration, intranasal administration has certain advantages. Intranasal administration is convenient and non-invasive, so it is suitable for preoperative induction anesthesia, sedation and analgesia before invasive surgery, and separation anxiety in children before surgery. It is reported that dexmedetomidine was administrated by dropping the injection formulation of dexmedetomidine into the nose (Sun et al., 2023; Li et al., 2018; Wang et al., 2019; Li et al., 2022) or using an MAD device (Uusalo et al., 2020; Xie et al., 2017). In fact, injection may not be suitable for intranasal administration, because the pH of the injection or some excipients may cause chemical stimulation of the nasal mucosa. However, no subjects in our study reported nasal discomfort after using the nasal spray. Therefore, it is necessary to develop a nasal spray formulation of dexmedetomidine and popularize it in clinical practice.

This study had some limitations. First, although the Bispectral Index (BIS) is widely used in anesthesia evaluation (Tekeli et al., 2020; Kim et al., 2020; Park et al., 2016; Zhang et al., 2021), it was not regarded as an indicator of PD in this study. Because many operations in the study process, such as blood sampling and blood pressure measurement, would greatly interfere with the BIS, thus affecting the accuracy of the study results, therefore, BIS was not collected as one of the PD indicators. Second, this study was conducted in healthy subjects, and owing to the ethical considerations, no pain stimulation was given to evaluate the analgesic effect of dexmedetomidine. Third, Ramsay scores were collected from before administration to 3 h after administration, which reflected a good process of process of sedation deepening and recovery in the low-dose groups. However, in Groups E and F, it was observed that some subjects remained sleepy after 3 h, and the Ramsay score was still at a high level at 3 h (>3). Therefore, although an obvious dose-effect relationship can be seen by collecting 3 h’ Ramsay score, if the data were collected for a longer time, the pharmacodynamic effects will be described more comprehensively.

## Conclusion

Dexmedetomidine nasal spray had a good dose-exposure effect in healthy Chinese male and female subjects. According to the PK data of each dose group, the dose-effect relationship can be modeled, thus providing theoretical basis for the study in children. In addition, we have proven that dexmedetomidine nasal spray has good safety and tolerance in the dose range of 25−200 μg. After the administration of 75 μg or more of dexmedetomidine nasal spray, an ideal sedation state could be achieved. Therefore, according to the comprehensive consideration of efficacy and safety, a dosage of 75−125 μg is recommended for phase II trials.

## Data Availability

The original contributions presented in the study are included in the article/Supplementary Material, further inquiries can be directed to the corresponding author.
