# Supplementary material for: Pharmacokinetics, pharmacodynamics and bioavailability of dexmedetomidine nasal spray in healthy Chinese adults: A phase I clinical trial
Source: Front Pharmacol. 2024 Nov 29;15:1488462. doi: 10.3389/fphar.2024.1488462 (PMC11638745; doi:10.3389/fphar.2024.1488462)
Supplement: Supplementary file 1 [file Table1.DOCX]

**Supplementary Table 1 Summarizes of the baseline characteristics of subjects**

| Parameters | Dexmedetomidine Group (n=67) | Placebo Group (n=14) | *P* |
| --- | --- | --- | --- |
| Age (y) | 32.49±8.04 | 32.43±7.04 | 0.9780 |
| Height (cm) | 165.26±6.63 | 164.61±5.88 | 0.7387 |
| Weight (Kg) | 61.28±6.08 | 61.85±7.25 | 0.7599 |
| BMI (kg/m^2^) | 22.44±1.79 | 22.79±2.03 | 0.5175 |

**Supplementary Table 2 Summary of adverse events**

| **Adverse events** | **Dexmedetomidine Nasal spray Group (n=67)** | | | **Placebo Nasal spray Group (n=14)** | | | **Dexmedetomidine IV Group (n=12)** | | | **Placebo IV Group (n=4)** | | |
| --- | --- | --- | --- | --- | --- | --- | --- | --- | --- | --- | --- | --- |
|  | **Cases** | **Subjects** | **Incidence rate(%)** | **Cases** | **Subjects** | **Incidence rate(%)** | **Cases** | **Subjects** | **Incidence rate(%)** | **Cases** | **Subjects** | **Incidence rate(%)** |
| Adverse events | 237 | 65 | 97.01 | 22 | 12 | 85.71 | 35 | 11 | 91.67 | 6 | 3 | 75.00 |
| Adverse reactions | 231 | 65 | 97.01 | 22 | 12 | 85.71 | 34 | 11 | 91.67 | 5 | 3 | 75.00 |
| SAEs | 0 | 0 | 0 | 0 | 0 | 0 | 0 | 0 | 0 | 0 | 0 | 0 |
| Adverse events leading to withdraw | 0 | 0 | 0 | 0 | 0 | 0 | 0 | 0 | 0 | 0 | 0 | 0 |

**Supplementary Table 3 The occurrence and comparison of various types of adverse reactions (Nasal spray group)**

|  | **Dexmedetomidine Nasal spray Group** | | | **Placebo Nasal spray Group** | | | **CMH test** |
| --- | --- | --- | --- | --- | --- | --- | --- |
|  | **Cases** | **Subjects** | **Incidence rate(%)** | **Cases** | **Subjects** | **Incidence rate(%)** | ***P*** |
| Investigations | 151 | 65 | 97.01 | 17 | 12 | 85.71 | 0.0777 |
| Slow heart rate | 58 | 58 | 86.57 | 7 | 7 | 50.00 | 0.0019 |
| Low blood pressure | 36 | 36 | 53.73 | 0 | 0 | 0.00 | 0.0003 |
| Extended QT interval of ECG | 21 | 21 | 31.34 | 0 | 0 | 0.00 | 0.0156 |
| Low diastolic blood pressure | 12 | 12 | 17.91 | 3 | 3 | 21.43 | 0.7594 |
| Low systolic blood pressure | 4 | 4 | 5.97 | 0 | 0 | 0.00 | 0.3514 |
| Elevated conjugated bilirubin | 3 | 3 | 4.48 | 0 | 0 | 0.00 | 0.4226 |
| Elevated urinary leukocyte | 1 | 1 | 1.49 | 2 | 2 | 14.29 | 0.0220 |
| High systolic blood pressure | 3 | 3 | 4.48 | 0 | 0 | 0.00 | 0.4226 |
| Elevated urinary erythrocyte | 1 | 1 | 1.49 | 1 | 1 | 7.14 | 0.2182 |
| Elevated diastolic blood pressure | 2 | 2 | 2.99 | 0 | 0 | 0.00 | 0.5153 |
| Extended QRS complex on ECG | 2 | 2 | 2.99 | 0 | 0 | 0.00 | 0.5153 |
| Rapid heart rate | 1 | 1 | 1.49 | 1 | 1 | 7.14 | 0.2182 |
| Elevated blood potassium | 2 | 2 | 2.99 | 0 | 0 | 0.00 | 0.5153 |
| Reduced percentage of monocytes | 0 | 0 | 0.00 | 1 | 1 | 7.14 | 0.0287 |
| Elevated low-density lipoprotein | 0 | 0 | 0.00 | 1 | 1 | 7.14 | 0.0287 |
| Hypothermia | 1 | 1 | 1.49 | 0 | 0 | 0.00 | 0.6476 |
| ECG PR shortening | 1 | 1 | 1.49 | 0 | 0 | 0.00 | 0.6476 |
| Abnormal T-wave of ECG | 1 | 1 | 1.49 | 0 | 0 | 0.00 | 0.6476 |
| Hypokalemia | 1 | 1 | 1.49 | 0 | 0 | 0.00 | 0.6476 |
| High blood pressure | 0 | 0 | 0.00 | 1 | 1 | 7.14 | 0.0287 |
| Reduced neutrophil count | 1 | 1 | 1.49 | 0 | 0 | 0.00 | 0.6476 |
| Cardiovascular disorders | 53 | 51 | 76.12 | 5 | 5 | 35.71 | 0.0031 |
| Sinus bradycardia | 51 | 50 | 74.63 | 5 | 5 | 35.71 | 0.0048 |
| I° atrioventricular block | 2 | 2 | 2.99 | 0 | 0 | 0.00 | 0.5153 |
| Gastrointestinal system disorders | 16 | 11 | 16.42 | 0 | 0 | 0.00 | 0.1051 |
| Nausea | 9 | 9 | 13.43 | 0 | 0 | 0.00 | 0.1483 |
| Vomiting | 5 | 5 | 7.46 | 0 | 0 | 0.00 | 0.2943 |
| Abdominal pain | 1 | 1 | 1.49 | 0 | 0 | 0.00 | 0.6476 |
| Xerostomia | 1 | 1 | 1.49 | 0 | 0 | 0.00 | 0.6476 |
| Nervous system disorders | 6 | 6 | 8.96 | 0 | 0 | 0.00 | 0.2475 |
| Dizziness | 5 | 5 | 7.46 | 0 | 0 | 0.00 | 0.2943 |
| Syncope | 1 | 1 | 1.49 | 0 | 0 | 0.00 | 0.6476 |
| Respiratory, thoracic, and mediastinal disorders | 2 | 2 | 2.99 | 0 | 0 | 0.00 | 0.5153 |
| Nasal discomfort | 1 | 1 | 1.49 | 0 | 0 | 0.00 | 0.6476 |
| Hypoxia | 1 | 1 | 1.49 | 0 | 0 | 0.00 | 0.6476 |
| Systemic diseases and various reactions at the administration site | 2 | 2 | 2.99 | 0 | 0 | 0.00 | 0.5153 |
| Fatigue | 2 | 2 | 2.99 | 0 | 0 | 0.00 | 0.5153 |
| Skin and subcutaneous tissue disorders | 1 | 1 | 1.49 | 0 | 0 | 0.00 | 0.6476 |
| Pruritus | 1 | 1 | 1.49 | 0 | 0 | 0.00 | 0.6476 |

**Supplementary Table 4 The occurrence and comparison of various types of adverse reactions (IV group)**

|  | **Dexmedetomidine Nasal spray Group** | | | **Placebo Nasal spray Group** | | | **CMH test** |
| --- | --- | --- | --- | --- | --- | --- | --- |
|  | **Cases** | **Subjects** | **Incidence rate(%)** | **Cases** | **Subjects** | **Incidence rate(%)** | ***P*** |
| Investigations | 21 | 11 | 91.67 | 4 | 3 | 75.00 | 0.3980 |
| Slow heart rate | 9 | 9 | 75.00 | 2 | 2 | 50.00 | 0.3657 |
| Low blood pressure | 7 | 7 | 58.33 | 0 | 0 | 0.00 | 0.0486 |
| Extended QT interval of ECG | 2 | 2 | 16.67 | 0 | 0 | 0.00 | 0.3980 |
| Elevated urinary leukocyte | 1 | 1 | 8.33 | 0 | 0 | 0.00 | 0.5637 |
| Hematuria | 0 | 0 | 0.00 | 1 | 1 | 25.00 | 0.0833 |
| Low systolic blood pressure | 1 | 1 | 8.33 | 0 | 0 | 0.00 | 0.5637 |
| Low diastolic blood pressure | 1 | 1 | 8.33 | 0 | 0 | 0.00 | 0.5637 |
| High diastolic blood pressure | 0 | 0 | 0.00 | 1 | 1 | 25.00 | 0.0833 |
| Cardiovascular disorders | 7 | 7 | 58.33 | 2 | 2 | 50.00 | 0.7782 |
| Sinus bradycardia | 7 | 7 | 58.33 | 2 | 2 | 50.00 | 0.7782 |
| Gastrointestinal system disorders | 4 | 3 | 25.00 | 0 | 0 | 0.00 | 0.2827 |
| Nausea | 3 | 3 | 25.00 | 0 | 0 | 0.00 | 0.2827 |
| Vomiting | 1 | 1 | 8.33 | 0 | 0 | 0.00 | 0.5637 |
| Systemic diseases and various reactions at the administration site | 3 | 3 | 25.00 | 0 | 0 | 0.00 | 0.2827 |
| Chest discomfort | 3 | 3 | 25.00 | 0 | 0 | 0.00 | 0.2827 |
