# Supplementary figures and images for: Pharmacokinetics, pharmacodynamics and bioavailability of dexmedetomidine nasal spray in healthy Chinese adults: A phase I clinical trial
Source: Front Pharmacol. 2024 Nov 29;15:1488462. doi: 10.3389/fphar.2024.1488462 (PMC11638745; doi:10.3389/fphar.2024.1488462)

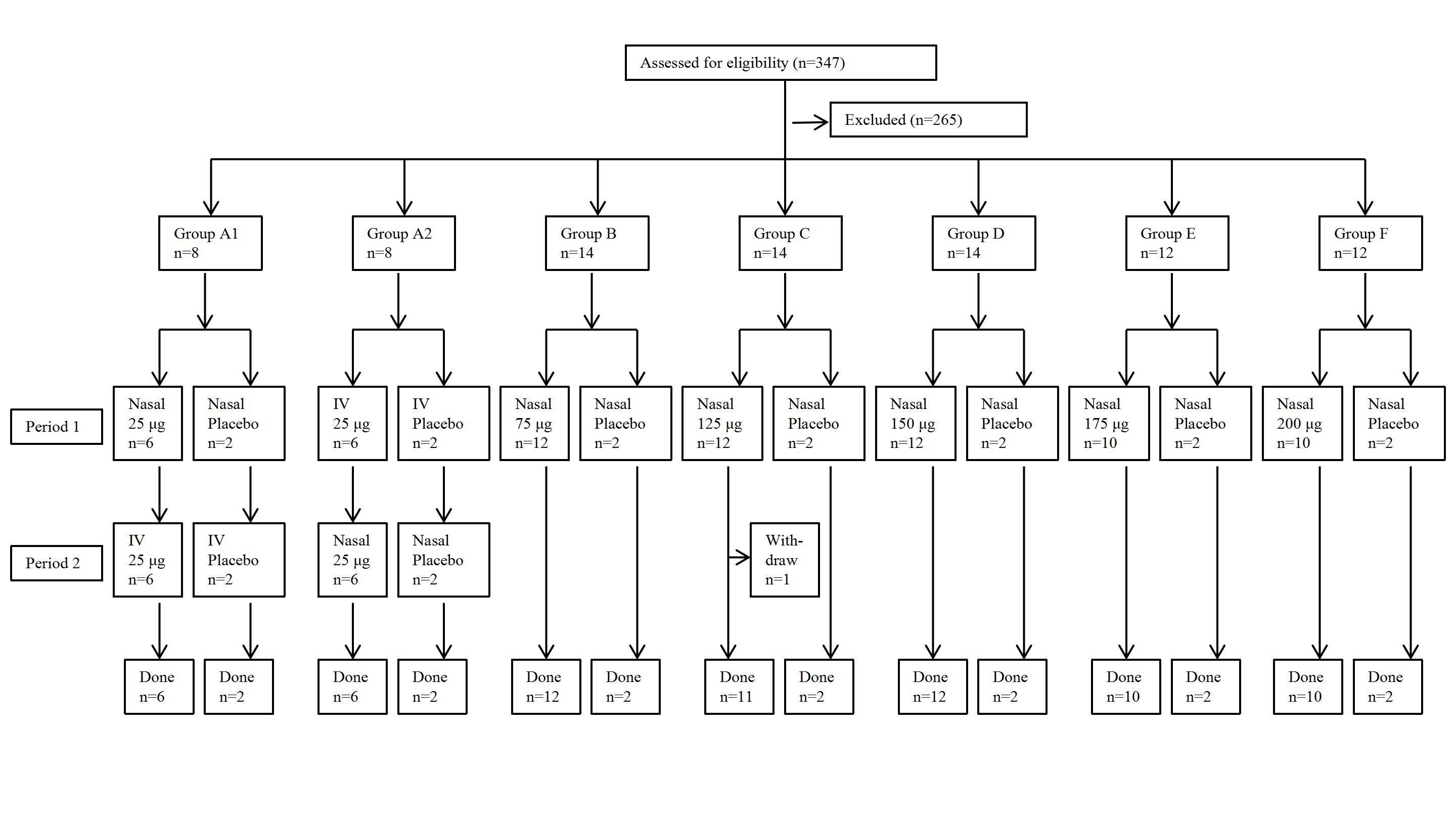

Supplement: Supplementary file 2 [file Image1.JPEG]
